# Plant debris are hotbeds for pathogenic bacteria on recreational sandy beaches

**DOI:** 10.1038/s41598-021-91066-w

**Published:** 2021-06-01

**Authors:** Yoshihiro Suzuki, Hiroki Shimizu, Takahiro Kuroda, Yusuke Takada, Kei Nukazawa

**Affiliations:** Department of Civil and Environmental Engineering, Faculty of Engineering, University of Miyazaki, Gakuen Kibanadai-Nishi 1-1, Miyazaki, 889-2192 Japan

**Keywords:** Microbiology, Environmental sciences

## Abstract

On recreational sandy beaches, there are guidelines for the management of bacterial pollution in coastal waters regarding untreated sewage, urban wastewater, and industrial wastewater. However, terrestrial plant debris on coastal beaches can be abundant especially after floods and whilst it has rarely been considered a concern, the bacterial population associated with this type of pollution from the viewpoint of public health has not been adequately assessed. In this study, microbes associated with plant debris drifting onto Kizaki Beach in Japan were monitored for 8 months throughout the rainy season, summer, typhoon season, and winter. Here we show that faecal-indicator bacteria in the plant debris and sand under the debris were significantly higher than the number of faecal bacteria in the sand after a 2015 typhoon. When we focused on specific pathogenic bacteria, *Brevundimonas vesicularis* and *Pseudomonas alcaligenes* were commonly detected only in the plant debris and sand under the debris during the survey period. The prompt removal of plant debris would therefore help create safer beaches.

## Introduction

Beaches are valuable recreational areas. They are also a tourism resource and the areas surrounding the locations of beaches are economically important. In order to promote the sustainable development of beaches and surrounding areas, an international system, “The Blue Flag Programme (BF)”, has been established by the Foundation for Environmental Education^1^, and the certification of beaches is rapidly spreading to various countries around the world. The BF consists of four categories (water quality, environmental management, environmental education, safety), of which water quality is an important category and requires the monitoring of coastal water quality. In particular, the number of fecal indicator bacteria in coastal waters due to the inflow of industrial wastewater and sewage is very important, and should be closely monitored when flooding occurs during torrential rain events. In contrast, standards have not yet been set for the quality of the beach sand that users come into direct contact with since information on the effects of bacterial contamination in beach sand is also important.

**Figure 1:**
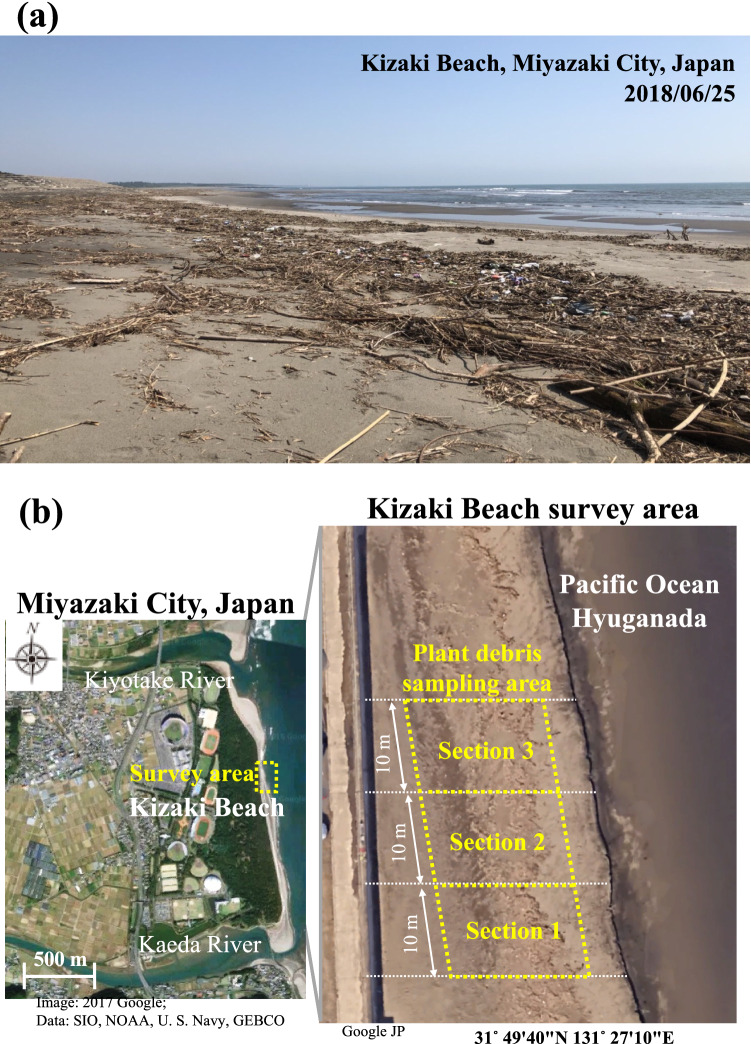
(**a**) Plant debris on Kizaki Beach on June 25, 2018. (**b**) Locations of sampling points on Kizaki beach, Miyazaki City, Japan. Most of the plant debris collected in this survey which accumulated near the high-water line of Kizaki Beach were branches and withered grass. All the plant debris was collected from three consecutive sections (Sections 1–3) along the high-water line at 10 m intervals (**b**) obtained from Google Earth (https://earth.google.com/web/@31.82602436,131.4436947,5.75042276a,5202.77770972d,35y,0h,0t,0r: https://earth.google.com/web/@31.82805704,131.45286349,1.42880734a,100.50282223d,35y,0h,0t,0r) and explanation and illustration were drawn in Microsoft Power Point (Version 16.16.27).

An enormous amount of terrestrial plant debris accumulates during heavy rainfall events such as mature cyclones (i.e., typhoons) and washes ashore on a coastal sand beach in the Pacific Ocean Hyuganada, which is directly affected by flooding from river, used for various recreational activities such as sea bathing, surfing, and sightseeing (Fig. 1a). Herein, we focused on the terrestrial plant debris that flowed outward from the land and accumulated on a coastal beach. Since plants are natural in origin the general public does not perceive any threat. Consequently, information on coastal plant debris is extremely scarce compared to marine plastic debris. Since plant debris has high water retention and a large specific surface area, it is very important from the viewpoint of a microbial habitat and may be a great concern with regard to public health, especially at recreational beaches. In addition to the inflow of treated sewage and wastewater onto the beach due to flooding, the drifting and accumulation of plant debris also causes bacterial contamination in beach sand and increases the health risk for beach users. Intervention by managers and care by users are required for sound management and usage based on hazards and risks as related to sandy beaches^2^. Beach management should also consider the threat to users due to sand contamination by bacteria.

Bacterial contamination of sandy beaches, although not well known, is extremely important from a public health perspective. Sand pollution is recognised as a significant risk to public health^3–10^. Faecal bacteria can accumulate and survive for long periods of time in sand and their concentrations can be 10 to 100 times greater than those in beach water^11–15^. In addition, after a typhoon event, heterotrophic bacteria, coliform bacteria, *Escherichia coli*, and enterococci can be 10–10^3^ times higher than what is normally detected in beach sand outside the water line^15^. The risk of beach users contracting an infectious disease can be strongly associated with the presence of pathogenic bacteria in the sand^11,16–21^. In addition to the inflow of sewage or drainage, plant debris is another likely source of bacterial contamination on beaches. For instance, the same genotype of Enterococci identified in river water has been detected in beach seawater after flooding^22^. Therefore, it is possible that terrestrial plant debris are exposed to treated sewage and/or drainage that flows outward at the time of flooding, and that fecal bacteria remain attached to drifting plant debris and accumulate on the beach. However, no study to date has investigated bacterial contamination associated with terrestrial plant debris drifting onto beaches based on continuous monitoring and bacterial florae analysis.

As a model area for this study, we evaluated Kizaki Beach, which faces the Hyuganada Sea in the Pacific Ocean and borders the Kyushu District in Japan, and is an important recreational base where the 2019 International Surfing Association World Surfing Games were held. Kizaki Beach is primarily intended for recreation by beach users and is not a major area for functioning conservation of natural beach ecosystems. The amount of plant debris drifting onto the beach and the number of faecal-indicator bacteria in the plant debris and the sand below the plant debris were monitored. Detection of pathogenic bacteria was attempted for each sample using bacterial florae analysis targeting the 16S rRNA gene. Additionally, laboratory experiments examined the survival of bacteria due to drought stress of plant debris and regrowth after the addition of water to simulate the conditions of dry weather and rainfall.

## Results

### Amount of plant debris on Kizaki Beach

**Figure 2:**
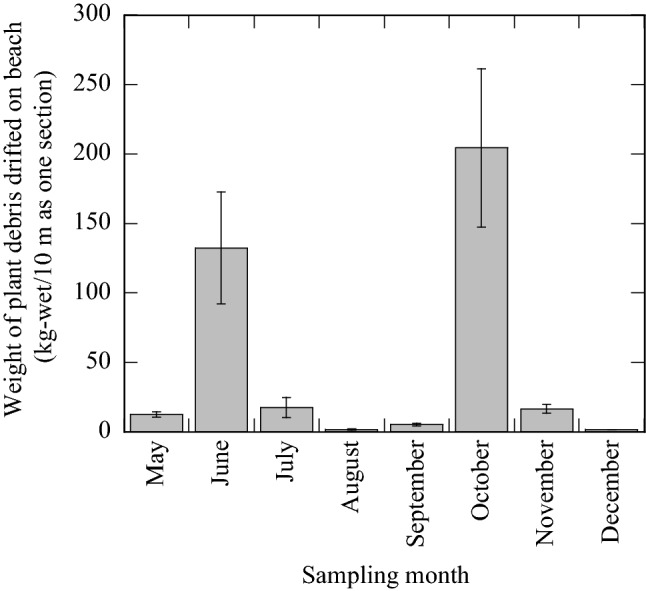
The monthly weight of plant debris accumulated on sections of Kizaki beach. Data shows the mean of three consecutive sections and error bars represent standard deviations (SD).

The locations and weight of plant debris in three 10 m sections of beach front in relation to the high-water line measured monthly are shown in Figs. 1b and 2. The amount of plant debris varied depending on the survey date. On June 25, 2018, the debris weight was 10 times larger (average 132.4 kg/10 m) than that on May 24. Flooding triggered by consecutive heavy rainfalls for three days (92.5 mm/day) presumably transported a large amount of plant debris from the watersheds of the Kiyotake and Kaeda Rivers before the survey in June. The debris weight on October 10 was 40 times larger (204.6 kg/10 m) than that on September 26 (5.3 kg/10 m). During this period, a September 28 typhoon caused severe flooding with localised heavy rain beginning October 4 (precipitation, 92.5 mm/day, rainfall intensity 34.0 mm/h). The amount of plant debris that drifted onto Kizaki Beach varied from 5 to 200 kg over 10 m up to the high-water line due to flooding caused by the rainfall. Regardless of the survey month, the major plants composing the debris were *Phragmites spp*., *Phyllostachys spp*., and *Ambrosia spp*., which are common to the floodplains of Japan. Kizaki beach is a straight, shallow, sandy beach, and debris of coastal plant products such as seaweed or seagrass are rarely washed ashore. Seaweed and seagrass were not present in the samples of plant debris in this survey.

The changes in water content and temperature of the plant debris and sand under the debris during the survey period are shown in Supplementary Figure S1. The water content of the debris varied greatly (range 8.2–31.3%). It was lowest in midsummer (August) when there was no rainfall. The water content of the sand just under the debris was stable and was within the range of 3.2–6.3% (average 4.6 ± 1.3%). The temperature of the sand was also stable at 36 °C in the summer season (July to September). It is assumed that the water content and temperature of the sand was stable because it was covered with debris.

### Bacterial pollution of the beach sand

**Figure 3:**
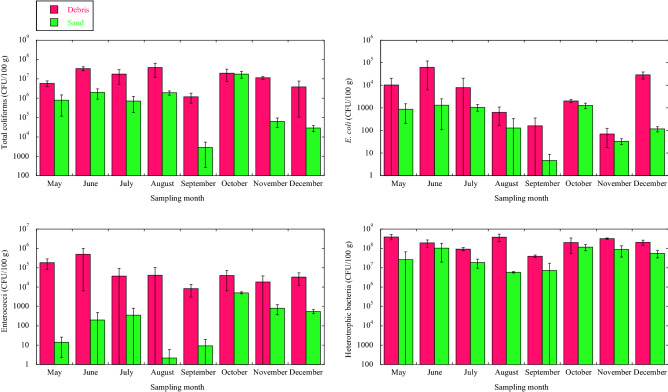
The changes in the numbers of total coliforms, *E. coli*, enterococci, and heterotrophic bacteria in the plant debris and the sand under the debris, during the entire survey period. Data are expressed as means ± standard deviations. ND, none detected.

The changes in the numbers of total coliforms, *E. coli*, enterococci, and heterotrophic bacteria in the plant debris and the sand under the debris of the beach during the entire survey period are shown in Fig. 3. During the survey period, no association was found between the weight of plant debris which had drifted onto the beach and the numbers of bacteria. In all the bacterial parameters, the bacterial counts associated with the plant debris were the highest, followed by the sand under the debris. The high number of total coliforms were detected continuously in the plant debris, ranging from 1.2 × 10^5^ to 3.8 × 10^7^ CFU/100 g (average: 1.7 × 10^7^ CFU/100 g). In the sand under the plant debris, the count of total coliforms was large (range 2.8 × 10^3^–1.7 × 10^7^ CFU/100 g (average: 2.9 × 10^6^ CFU/100 g)). *E. coli,* as a noticeable parameter, was detected in high concentrations in the plant debris (1.0 × 10^4^ CFU/100 g on May 24, 6.3 × 10^4^ CFU/100 g on June 25). Although the total number of coliforms and *E. coli* in the sand under the plant debris was lower than that in the debris, the change in counts tended to follow the amount of plant debris. In contrast, the change of the number of enterococci in the debris was different from the sand. The enterococci count in the plant debris was markedly high on May 24, June 25, August 30, and September 26 (ranging from 8.3 × 10^3^ to 5.0 × 10^5^ CFU/100 g); more than 10^3^ times greater than in the sand under the debris. On the other hand, the number of heterotrophic bacteria in the debris and sand was high and fluctuated to a lesser extent ranging from 5.2 × 10^7^ in the sand to 2.3 × 10^8^ CFU/100 g in the debris on average.

### Comparison of the bacterial Genera between plant debris and sand

**Figure 4:**
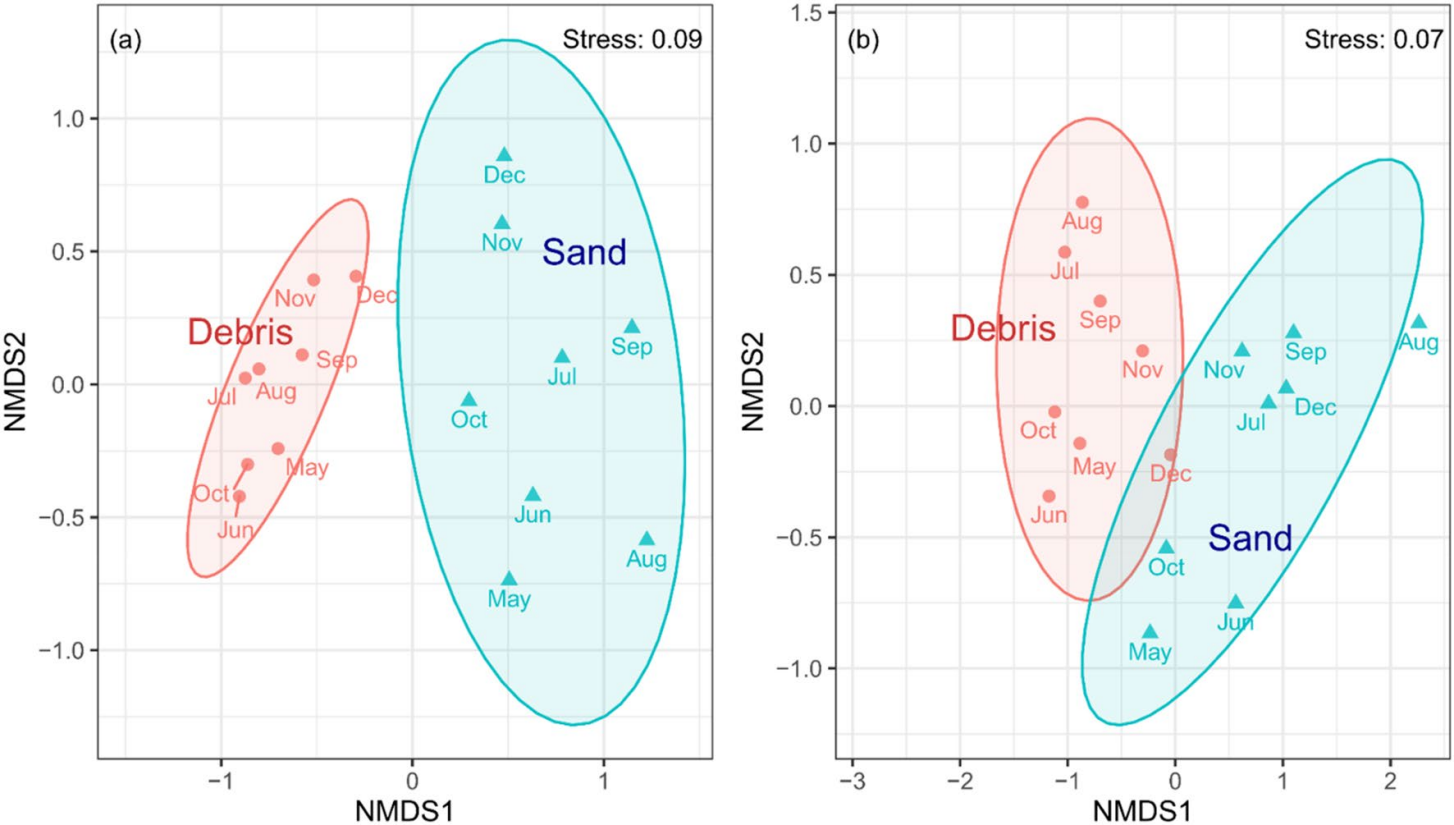
Non-metric multidimensional scaling with Bray–Curtis distance based on relative abundances of all bacterial Genera: (**a**) and 84 pathogenic bacterial Genera; (**b**) in debris (circle) and sand under the debris (triangle) from May to December. Ellipses surrounding each environmental group indicate 90% confidence levels.

The samples of plant debris and sand under the debris averaged 23,834 ± 4166, (24 samples: 3 sections and 8 months) and 22,120 ± 4229 (24 samples: 3 sections and 8 months) operational taxonomic units (OTUs), respectively, according to next-generation sequencing results. We identified 1249, and 1989 bacterial Genera in the samples of plant debris and sand under the debris, respectively. Based on the OTU results of non-metric multi-dimensional scaling (NMDS), the bacterial communities were clearly segregated by plant debris and sand under the debris (Fig. 4). Figure 4a depicts the relative abundances of all bacterial Genera, and Fig. 4b includes only pathogenic bacterial Genera. In each sample, the total bacterial flora showed independent construction (Fig. 4a).

**Figure 5:**
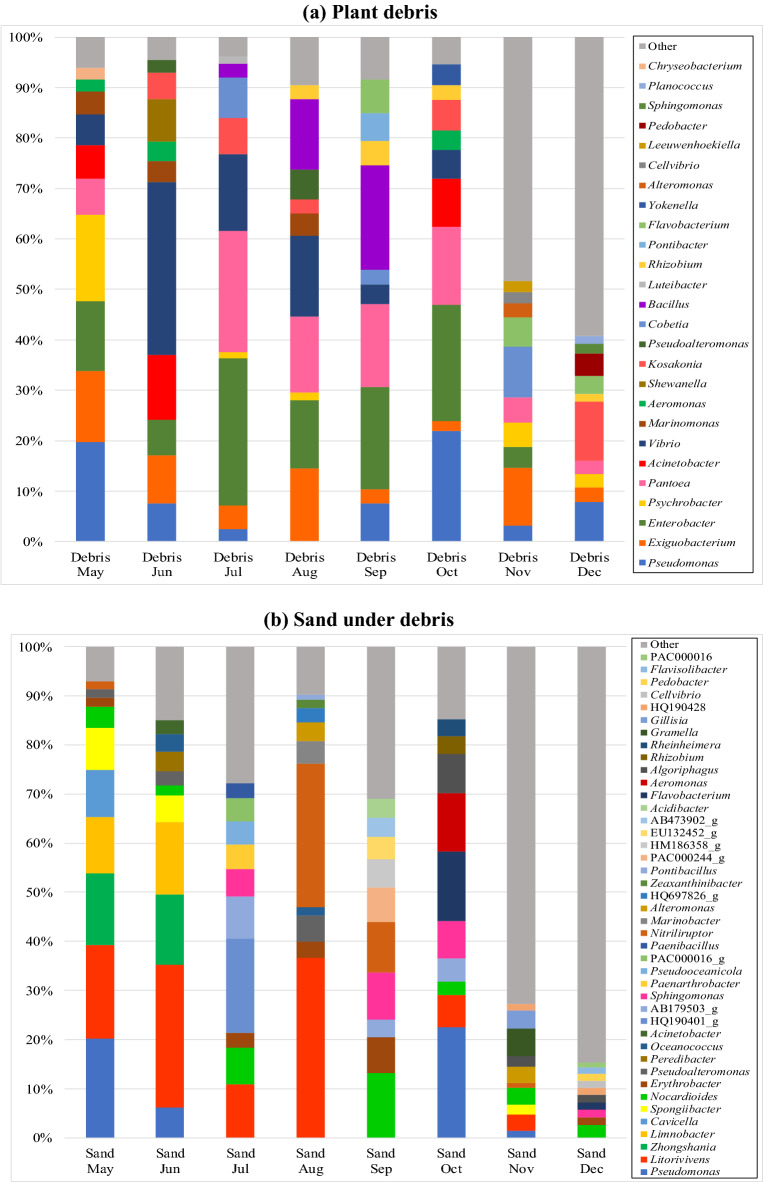
The distributions of the 10 most abundant Genera in these sample sets.

The distributions of the 10 most abundant Genera in these samples are shown in Fig. 5a. The Genera displayed variety and changed each month due to changes in environmental conditions such as water content and temperature. The major Genera detected in the plant debris throughout the survey period were: *Enterobacter* (4.1–29.2%), *Vibrio* (3.7–34.2%), and *Pantoea* (2.6–24.0%). The changes in the distributions of the 10 most abundant orders in the beach sand under the debris are shown in Fig. 5b with the major Genera throughout the survey being *Litorivivens* (3.4–36.6%), *Pseudomonas* (1.4–22.5%), and *Nocardioides* (2.6–13.2%).

### Variation of potential pathogen bacteria

The pathogens that posed potential risks to humans were extracted from the OTU datasets based on the list of pathogenic bacterial Genera presented by Fang et al.^23^. Eighty-four of the 145 Genera in the pathogenic bacterial list were detected. Based on the NMDS analysis of potential pathogenic bacteria, the 90% confidence level debris cluster partially overlapped with the sand under the debris cluster suggesting a partial relationship between these two bacterial groups (Fig. 4b). From a comprehensive view of the NMDS analysis for potential pathogenic bacterial Genera, it can be inferred that the plant debris and sand under the debris form distinctive community structures, but partly depend on each other based on physical characteristics among the environments.

A total of 35 major pathogenic bacteria with an abundance ratio (OTU number of each bacterium/OTU number of all bacteria) of 0.1% or more were detected. *Bacillus, Mycobacterium, Pseudomonas*, and *Sphingomonas* were detected in the samples during the survey period (Supplementary Figure S2). Permutational multivariate analysis of variance (PERMANOVA) revealed significant differences among the plant debris and sand under the debris (*p* < 0.01). The prevalence of potential pathogenic bacteria in the plant debris is significantly high than the sand under the debris. The debris samples showed a greater difference when compared to the sand under the debris based on adjusted *p* values (< 0.001). *Enterobacter, Vibrio, Pantoea*, and *Pseudomonas* were detected at relatively high concentrations in the plant debris, and the average abundances were 10.1%, 8.23%, 8.16%, and 7.02%, respectively. *Pseudomonas, Sphingomonas, Aeromonas*, and *Bacillus* were detected at relatively high concentrations in the beach sand just under the plant debris with the average abundances being 3.77%, 1.46%, 0.91%, and 0.48%, respectively. It was characteristic that *Enterobacter*, an attenuated bacterium of the *Enterobacteriaceae* family, was detected in more than 10% of the debris samples, and that the potentially pathogenic Genus *Pseudomonas,* was mainly detected in both debris and sand under the debris. *Vibrio* is a common bacterial Genus in coastal substrates^15^. These results suggest that plant debris has the highest potential to hold pathogenic bacteria on sandy beaches.

### Change in the bacterial count and flora under drying and wetting conditions

**Figure 6:**
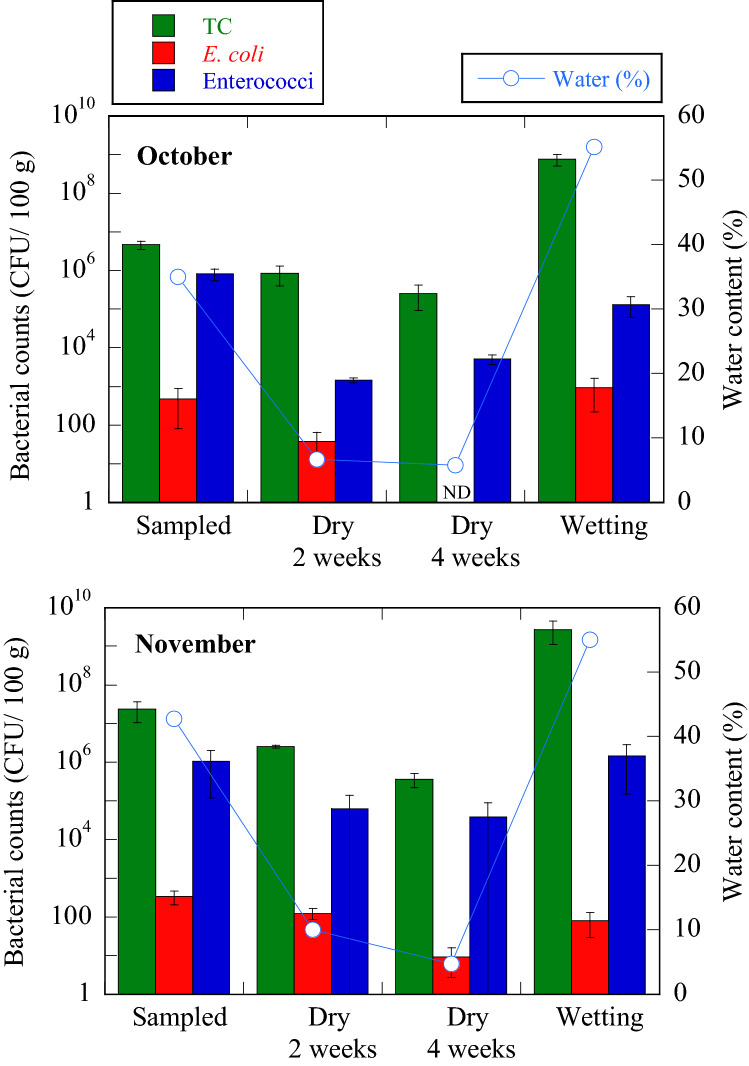
The change in the bacterial count of plant debris under drying and wetting conditions. The plant debris used in the drying-wetting experiment was collected from Kizaki Beach. The raw plant debris were dried for 4 weeks in the dark at 37 °C and then immersed in sterile distilled water for 1 h. The wet plant debris were then incubated at 30 °C for 24 h. TC, total coliforms, ND, not detected, CFU, colony forming units.

The changes in the bacterial counts of plant debris under drying and wetting conditions are shown in Fig. 6. Immediately after harvesting debris from the beach in October 2019, the number of total coliforms was found to be 4.6 × 10^6^ CFU/100 g with a 35% water content. After drying for 2 weeks, the water content of the plant debris dropped significantly to 6.7%, and the number of total coliforms decreased to 8.3 × 10^5^ CFU/100 g. The dried debris was in a ‘dry state’ with no visible moisture. Four weeks later, the water content had decreased to 5.8% and the number of total coliforms remained relatively unchanged (10^5^ CFU/100 g). When the dried debris were soaked in sterile distilled water and incubated for 24 h (water content, 55.2%), the number of total coliforms drastically increased to 7.6 × 10^8^ CFU/100 g. *E. coli,* which had been undetectable after 4 weeks of drying, was detected at 9.3 × 10^2^ CFU/100 g after wetting. The number of enterococci which had decreased from 8.2 × 10^5^ to 1.5 × 10^3^ CFU/100 g after 2 weeks of debris drying and which was detected at 5.1 × 10^3^ CFU/100 g after drying for 4 weeks, increased to 1.3 × 10^5^ CFU/100 g after wetting. In drying and wetting repeat experiments using different plant debris collected in November 2019, total coliforms and enterococci were active in the debris dried for 4 weeks at 3.6 × 10^5^ CFU/100 g and 3.9 × 10^4^ CFU/100 g, respectively with 4.7% wet content. In contrast, *E. coli* was affected by drying with counts decreasing from 3.4 × 10^2^ to 9.3 CFU/100 g after 4 weeks of drying, which then increased to 79 CFU/100 g after wetting.

As a result of the florae analysis of the plant debris at each stage of the two drying and wetting experiments, a total of 1249 bacterial Genera were identified from all the samples. Among the bacterial Genera detected in each sample, the top 10 Genera with the highest relative abundances are shown in Supplementary Figure S3. The bacterial florae of plant debris collected in October and November were quite different. However, *Bacillus*, *Erythrobacter*, *Rhizobium*, *Nocardiodes*, *Sphingomonas*, and *Novoshingobium* were commonly identified as the 10 major Genera under dry conditions. *Bacillus*, *Enterobacter*, and *Rhizobium*, which were detected in the just-collected debris, are extremely resistant to drought conditions, and their relative abundance in dried debris after 4 weeks was 5.2%, 4.4%, and 4.4%, respectively.

## Discussion

### Plant debris as bacterial pollution sources on sandy beaches

From the viewpoint of public health for beach users, BF requires that beach managers monitor the quality of coastal waters and post and publish the results^24^. As a source of water quality pollution, sewage and drainage of urban wastewater due to torrential rain and are the most important management items. Particular attention has been paid to faecal bacteria, and the limits for *E. coli* and enterococci in coastal water are strictly set at 250 CFU/100 ml and 100 CFU/100 ml, respectively. In order to protect the health of beach users, preventive measures must be taken to address the increased bacterial load after flooding events. On the other hand, although water quality standards have been set, standards or monitoring items related to sand quality have not. However, the accumulation of plant debris on beaches due to flooding has a large effect on the sand quality.

*E. coli* was detected in high concentrations in the plant debris with the average being 1.4 × 10^4^ CFU/100 g throughout the monitoring period. Furthermore, the average number of enterococci (1.1 × 10^5^ CFU/100 g) was 10 times greater than that of *E. coli* in the debris. The bacterial impact is significantly greater in the plant debris than what was detected after the typhoon. It can be assumed that 10^6^ CFU/100 g of faecal-indicator bacteria can be routinely detected from rotting plants and sand under the plants on most beaches. However, it has been reported that there is a trend of increasing risk for *Enterococcus* > 13 CFU/g (= 1.3 × 10^3^ CFU/100 g) in beach sand with enteric illnesses among beachgoers^20^. After evaluating the number of enterococci in each sample based on the 13 CFU/g level, all debris and the sand under the debris collected in October exceeded the level of increasing risk for *Enterococcus*. Therefore, leaving plant debris on recreational beaches may increase the risk of enteric illnesses for beach users.

When we focused on potentially pathogenic species, 44 among the list of 538 pathogenic bacterial species^27^, were extracted from the OTU data. In addition, the abundance ratio was 0.012 to 15.3% (Supplementary Figure S4). The highest ratio for the plant debris and sand under the debris were 6.25% in July and 0.79% and in October respectively. *Brevundimonas vesicularis* and *Pseudomonas alcaligenes* were commonly detected in the plant debris and sand under the debris during the survey period. *B. vesicularis* is widely known as a phytopathogenic bacteria. *Pseudomonas* spp. is a major component of plant root bacteria. Therefore, the route of transmitted *B. vesicularis* and *P. alcaligenes* to the sand would be via plant debris. The NMDS ordination (Fig. 4b), which shows the link between debris and sand, supports this hypothesis.

Based on the results of drying-wetting experiments of plant debris, we confirmed that total coliforms and enterococci survived at the range of 10^5^ to 10^6^ CFU/100 g and 10^3^ to 10^4^ CFU/100 g, respectively, even in very dry conditions (5% moisture content). In general, total coliforms include potentially pathogenic bacteria. It has been recognized that enterococci, which cause opportunistic infections, are detected at high concentrations in dry beach sand and have a high tolerance for dry conditions^25,26^. Therefore, plant debris will most likely be the main source of enterococci pollution on beaches as seen on Kizaki Beach. Although *E. coli* had a weaker resistance to drying than total coliforms and enterococci, *E. coli* regrew under wet conditions. Both *Bacillus* and *Erythrobacter* are included in the list of 145 bacterial Genera suspected of being pathogenic. Furthermore, the pathogenic bacteria *Bacillus pumilus* and *Pseudomonas alcaligenes* were detected in the dried debris. In both of the two experiments, even when the dried plant debris were returned to humid conditions, the debris showed completely different florae from when it was collected. However, *Pantoea* was a common Genus that proliferated in humid conditions. It was inferred that the florae associated with plant debris on sandy beaches changed due to repeated drying and wetting conditions.

There is a definite need to assess pollution on beaches in terms of human health and safety risks^2^. Our research can contribute to the provision of information related to establishing new risk assessments.

### Sustainable management for creating safe beaches

In 2009, Japan enacted policies which ensured the conservation of good coastal landscapes and environments through treatment of marine debris that drifted ashore and effectively reduced their generation. Coastal managers must take measures necessary for the treatment of marine debris to keep coastal land under their control clean in terms of landforms, landscapes, ecosystems, and other natural conditions, whilst considering the utilisation, economic activities, and other social opportunities of the coast. Many other countries have laws and/or regulations with similar purposes for marine debris. In the European Union (EU), the most important initiative to address marine litter is the Marine Strategy Framework Directive (MSFD) established in 2008. Leading the United States’ Federal Government's measures against marine debris is the Marine Debris Research, Prevention, and Reduction Act of 2006. Currently, the BF, as an international environmental certification, shows the criteria for solving problems related to beach environmental conservation and management including marine debris and beach waste^24^, and it is being promoted in various countries around the world, including Japan^1^.

Among the marine debris and beach waste, plant debris has a natural origin. Therefore, although the plant debris evaluated in this study are quantitatively more abundant than plastic debris on Kizaki Beach, the general public does not perceive any threat. However, as demonstrated in the present study, faecal-indicator bacteria as well as pathogenic bacteria were retained at high densities on the plant debris that drifted ashore with heavy rain and typhoon events, indicating that plant debris is a suitable habitat for such bacteria. The bacteria in plant debris are extremely resistant to drought, and can survive for 2–4 weeks with a water content of only 5%. Furthermore, it was also shown that even if the numbers of bacteria were reduced by drying, the bacteria re-grew after wetting. For recreational beach users, the drifting and accumulation of plant debris increases the risk of illness due to contact and infection by pathogenic bacteria at a high density.

Since beach conservation has a variety of targets and purposes regarding public health, ecosystem conservation, and landscape conservation, cleaning the plant debris on the beach is required for each purpose^28^.To reduce the health risks for users of recreational sandy beaches, plant debris should be given a high priority in maintenance management and should be considered a potential health hazard for infectious diseases, and not just harmless naturally-occurring products. Based on this study, we propose that to address the public health issues of beaches and coasts, it is necessary to collect and treat plant debris to the same extent as plastic marine debris since the prompt removal of plant debris can create safer recreational beaches. The coastal managers should monitor beaches and develop a plan for the collection of plant debris in the event of flood events which can occur just after heavy rain and typhoon events. On the other hand, beach management requires a budget, so it is important to discuss these issues with local users, tourists, and administrative managers. For the management of Kizaki Beach, a council of local governments, local contractors, and users has been established. We propose to consider the collection and adequate disposal of plant debris for the purpose of protecting the user’s health.

## Materials and methods

### Sampling sites

Kizaki Beach, one of the main recreational beaches in Japan, is situated between the outlets of the Kiyotake and Kaeda Rivers (Fig. 2). Kizaki Beach is a prefectural general sports park (total area 154 hectares) that has advanced facilities designed to respect the natural environment, so there are no houses, factories, or farms near the beach. In addition, there are no drain pipes connected to Kizaki Beach; therefore, there is no inflow from domestic or agricultural sources. The Kiyotake and Kaeda Rivers encompass 166.4 and 53.8 km^2^, respectively, and their corresponding lengths are 28.8 and 17.5 km, respectively. Although, there are small-scale sewage treatment plants along the Kiyotake River, they use a discrete and segregated system so sewage does not flow directly into the river even during heavy rains. Moreover, > 90% of the catchment areas of the Kaeda River are forests. Therefore, the coastal water around Kizaki Beach is classified as the highest quality by the Ministry of the Environment, Japan. Normally, the numbers of *E. coli* and enterococci in the coastal waters are 1 CFU/100 mL and 0–1 CFU/100 mL, respectively^15^. Topographically, since Kizaki Beach is linearly shallow for 2 km facing the Pacific Ocean, the beach is typically an open system. However, plant debris, which includes driftwood, branches, and withered grass, accumulates along the high-water line on Kizaki Beach. During large-scale rainfall events such as typhoons, a large amount of plant debris accumulates and seriously hinders the recreational use of this beach. Since it is frequently impacted by drifting plant debris, it was selected as the model beach for this study.

### Sampling of plant debris and beach sand

We selected the narrow area of plant debris washed ashore on the beach as the survey area (Fig. 1b), and focussed on the plant debris and the sand under the debris. To monitor the effects of plant debris on bacterial contamination of the beach, this survey was conducted monthly for eight months (May 24—December 18, 2018) at a fixed location and included the rainy season and the typhoon season from summer to autumn. The plant debris collected were ≤ 30 mm in diameter and ≤ 100 cm in length. Most of the plant debris accumulated near the high-water line on Kizaki Beach collected during this survey were branches and withered grass. Plant debris was collected in plastic bags from three consecutive sections (Sections 1, 2, and 3) along the high-water line at 10 m intervals. We assumed that the amount of collected debris was a rough standing stock estimate for each survey month. From the three sections where plant debris were collected, sand (surface layer − 10 cm) just under the plant debris was collected using an acrylic column. For each section, three points were randomly selected, and sand was composited to obtain each section sample.

The collected plant debris was taken back to the laboratory within 30 min after collection, and each 10 m sample section was measured for wet weight (kg wet/10 m as one section). This wet weight of plant debris was defined as the existing amount of plant debris per 10 m of section on each survey day. On June 25 and October 10, 2018, when it was difficult to collect the entire amount of plant debris which had drifted onto the beach, plant debris was collected within a range of 1–2 m along the high-water line and the weight was measured and then converted to a weight which represented 10 m. After measuring the weight, the plant debris was pulverized to a length of about 5 cm using a pulveriser (MGS-1510Si, Minato Electric Industries, Oita, Japan). The pulverized plant debris and sand were dried at 110 °C for 24 h, and the dry weight was measured. The water content (%) was determined from the wet and dry weight of each sample.

### Enumeration of faecal-indicator bacteria

Plant debris (300 g) was thoroughly mixed with 3 L of sterilised physiological saline solution in a sterilised 5 L polyethylene bottle for 2 min. Sand (5 g) was mixed for 2 min with 40 mL of sterilised physiological saline solution. After settling for 1 min, the supernate was aliquoted, and the extract was serially diluted. CHROMagar ECC agar plates (CHROMagar, Paris, France) were used to differentiate and count *Escherichia coli* and other coliform bacteria. The diluted supernate was filtered through a 0.45-μm pore membrane filter (47-mm diameter, sterile, mixed cellulose ester; Advantec, Tokyo, Japan) and incubated on ECC agar plates for 24 h at 37 °C. Blue colonies were identified as *E. coli* isolates. Mauve colonies were designated isolates of other coliform species. Enterococci were enumerated on membrane-Enterococcus indoxyl-β-d-glucoside (mEI) agar plates using the membrane filter method^29^. The diluted supernates were passed through a membrane filter and the filtrate was used to inoculate membrane-Enterococcus indoxyl-β-d-glucoside (mEI) agar plates for 24 h at 41 °C. Blue colonies were identified as enterococci isolates. Heterotrophic bacteria were enumerated on agar plates (agar 1.5%, Difco Marine Broth 2216; Becton, Dickinson and Company, MD, USA). The diluted supernate was spread onto the plates and incubated for 7 days at 22.5 °C. After incubation, colonies were counted as heterotrophic bacteria.

The number of coliform bacteria, *E. coli*, enterococci, and heterotrophic bacteria of all samples was determined from the mean number of colony-forming units (CFUs) of 3 replicates. The bacterial count was expressed as CFU/100 g of debris or sand. The detection limits of each bacterial count in the plant debris and sand samples were 3 × 10^1^ CFU/100 g, and 7 CFU/100 g, respectively.

The supernates extracted from the plant debris and sand were then filtered through a membrane filter. DNA was extracted from the bacteria collected on the membrane filters using a DNeasy PowerWater kit (QIAGEN, Hilden, Germany) following the manufacturer's instructions. The concentrations of DNA in the extracts were determined using a Quantus fluorometer (Promega, WI, USA) and an appropriate kit.

### 16S rRNA-gene sequencing and data analysis

The V3–4 region of the bacterial 16S rRNA genes of each sample were amplified using specific primers that included an overhang adapter sequence linked to the 5′ ends of the primers (forward primer, 5′-ACACTCTTTCCCTACACGACGCTCTTCCGATCT-NNNNN-CCTACGGGNGGCWGCAG-3′; and reverse primer, 5′-GTGACTGGAGTTCAGACGTGTGCTCTTCCGATCT-NNNNN-GACTACHVGGGTATCTAATCC-3′).

The polymerase chain reaction (PCR) reaction solution (total 10 µL) was mixed with 1.0 µL of 10 × Ex Taq Buffer (TaKaRa, Tokyo, Japan), 0.8 µL of dNTP Mixture (TaKaRa), 0.5 µL of each primer, 2.0 µL of Template DNA, and 0.1 µL of Ex Taq Hot Start Version (TaKaRa), and 5.1 µL of deionized distilled water (DDW). The PCR reactions were performed as follows: initial denaturation at 94 °C for 2 min, 30 cycles of denaturation at 94 °C, annealing at 55 °C for 30 s, extension at 72 °C for 30 s, and final extension at 72 °C for 5 min. The PCR products were purified using Agencourt AMPure (Beckman Coulter, CA, USA).

The purified PCR products were subjected to a second-round of PCR amplification with (2nd forward primer, 5′-AATGATACGGCGACCACCGAGATCTACAC-Index-ACACTCTTTCCCTACACGACGC-3′; and 2nd reverse primer, 5′-CAAGCAGAAGACGGCATACGAGAT-Index-GTGACTGGAGTTCAGACGTGTG-3′). The polymerase chain reaction (PCR) reaction solution (total 10 µL) was mixed with 1.0 µL of 10 × Ex Taq Buffer, 0.8 µL of dNTP Mixture, 0.5 µL of each primer, 2.0 µL of Template DNA, and 0.1 µL of Ex Taq Hot Start Version, and 5.1 µL of DDW. The PCR reactions were performed as follows: initial denaturation at 94 °C for 2 min, 10 cycles of denaturation at 94 °C, annealing at 60 °C for 30 s, extension at 72 °C for 30 s, and final extension at 72 °C for 5 min. The PCR products were purified using Agencourt AMPure, and sequenced 2 × 300-bp paired-end by the MiSeq platform (Illumina, Inc., CA, USA) using the MiSeq v3 Reagent Kit (Illumina). The sequencing data were analysed using Qiime ver. 1.9.0 (Pycogent, GitHub Inc., CA, USA). Raw paired-end reads were trimmed to remove low-quality sequences and then merged. All the sequences that were not determined to be chimeras were extracted and was performed to create Operational Taxonomic Units (OTUs) sequence. The merged reads were clustered into OTUs at 97% sequence identity based on the EzBioCloud 16S database. A representative sequence of each OTU was identified to the phylogenetic division or genus or species.

### Drying-wetting experiments of plant debris in an incubator

Plant debris that drifted to the shore presumably experienced repetitive drying and wetting depending on the weather. The number of bacteria attached to the debris must also be significantly affected by water content. Therefore, the drying condition of plant debris was artificially controlled in a laboratory incubator, and the inactivation and reactivation of bacteria by drying and wetting was investigated. The plant debris used in the drying-wetting experiment was collected from targeted areas on Kizaki Beach. The raw plant debris was dried for 4 weeks in the dark at 37 °C. Aliquots of the dried debris were sampled every two weeks. Furthermore, the plant debris dried for 4 weeks were immersed in sterile distilled water for 1 h. The wet plant debris were wrapped in aluminium foil to prevent evaporation and incubated at 30 °C for 24 h. For all the debris samples (3 aliquots for each sample): raw debris, 2- and 4-week dried debris, and re-wetted debris; the bacterial counts and water content were determined as described previously. In addition, DNA was also extracted from the debris samples for bacterial florae analyses. Drying-wetting experiments were repeated twice using different plant debris which were collected in October and November, 2019.

### Statistical analysis

Based on the relative abundances (OTU number) of the bacterial Genera determined by sequencing analysis, we derived a Bray–Curtis distance between the plant debris and sand samples among the sampling periods. The distances measured were subsequently used for performing non-metric multidimensional scaling (NMDS) using the metaMDS function from the vegan package (R v.3.6.3). Florae differences among the debris and sand samples were also tested using permutational multivariate analysis of variance (PERMANOVA) with the pairwise.adonis function in the pairwiseAdonis package in R ver. 3.6.3. The test used the Bray–Curtis distance and was based on 10,000 permutations. Subsequently, post-hoc pairwise comparisons of the similarity between the samples were performed with an adjusted *p* value based on the Bonferroni correction using the pairwise.adonis function in the pairwiseAdonis package (R v.3.6.3).

## Supplementary information

Supplementary Information.
