# Supplementary material for: Plant debris are hotbeds for pathogenic bacteria on recreational sandy beaches
Source: Sci Rep. 2021 Jun 1;11:11496. doi: 10.1038/s41598-021-91066-w (PMC8169675; doi:10.1038/s41598-021-91066-w)
Supplement: Supplementary file 1 — Supplementary Information. [file 41598_2021_91066_MOESM1_ESM.docx]

**Plant debris are hotbeds for pathogenic bacteria on recreational sandy beaches**

Yoshihiro Suzuki*, Hiroki Shimizu, Takahiro Kuroda, Yusuke Takada, Kei Nukazawa

Department of Civil and Environmental Engineering, Faculty of Engineering, University of Miyazaki, Gakuen Kibanadai-Nishi 1-1, Miyazaki 889-2192, Japan.

*Corresponding author:
Yoshihiro Suzuki

Department of Civil and Environmental Engineering, Faculty of Engineering, University of Miyazaki, Gakuen Kibanadai-Nishi 1-1, Miyazaki 889-2192, Japan

Phone No: +81-985-58-7339

Fax No: +81-985-58-7344

[ysuzuki@cc.miyazaki-u.ac.jp](mailto:ysuzuki@cc.miyazaki-u.ac.jp)

**Supplemental Figure Caption**

Supplementary Figure S1. The changes in water content and temperature of plant debris and sand during the survey period.

Supplementary Figure S2. Heatmap of the relative abundances (> 0.1%) of 35 genera detected as human pathogenic bacteria in the total sequences of each sample.

Supplementary Figure S3. The distributions of the 10 most abundant Genera in each sample in drying and wetting conditions.

Supplementary Figure S4. Heatmap of the relative abundances (> 0.1%) of 9 species detected as human pathogenic bacteria in the total sequences of each sample.

Supplementary Figure S1. The changes in water content and temperature of plant debris and sand under the debris during the survey period.

Supplementary Figure S2. Heatmap of the relative abundances (> 0.1%) of 35 genera detected as human pathogenic bacteria in the total sequences of each sample.

Supplementary Figure S3. The distributions of the 10 most abundant Genera in each sample in drying and wetting conditions.

Supplementary Figure S4. Heatmap of the relative abundances (> 0.1%) of 9 species detected as human pathogenic bacteria in the total sequences of each sample.
